# Combined Analyses of the ITS Loci and the Corresponding 16S rRNA Genes Reveal High Micro- and Macrodiversity of SAR11 Populations in the Red Sea

**DOI:** 10.1371/journal.pone.0050274

**Published:** 2012-11-20

**Authors:** David Kamanda Ngugi, Ulrich Stingl

**Affiliations:** Red Sea Research Center, King Abdullah University of Science and Technology (KAUST), Thuwal, Saudi Arabia; Universidad Miguel Hernandez, Spain

## Abstract

Bacteria belonging to the SAR11 clade are among the most abundant prokaryotes in the pelagic zone of the ocean. 16S rRNA gene-based analyses indicate that they constitute up to 60% of the bacterioplankton community in the surface waters of the Red Sea. This extremely oligotrophic water body is further characterized by an epipelagic zone, which has a temperature above 24°C throughout the year, and a remarkable uniform temperature (∼22°C) and salinity (∼41 psu) from the mixed layer (∼200 m) to the bottom at over 2000 m depth. Despite these conditions that set it apart from other marine environments, the microbiology of this ecosystem is still vastly understudied. Prompted by the limited phylogenetic resolution of the 16S rRNA gene, we extended our previous study by sequencing the internal transcribed spacer (ITS) region of SAR11 in different depths of the Red Sea’s water column together with the respective 16S fragment. The overall diversity captured by the ITS loci was ten times higher than that of the corresponding 16S rRNA genes. Moreover, species estimates based on the ITS showed a highly diverse population of SAR11 in the mixed layer that became diminished in deep isothermal waters, which was in contrast to results of the related 16S rRNA genes. While the 16S rRNA gene-based sequences clustered into three phylogenetic subgroups, the related ITS fragments fell into several phylotypes that showed clear depth-dependent shifts in relative abundances. Blast-based analyses not only documented the observed vertical partitioning and universal co-occurrence of specific phylotypes in five other distinct oceanic provinces, but also highlighted the influence of ecosystem-specific traits (e.g., temperature, nutrient availability, and concentration of dissolved oxygen) on the population dynamics of this ubiquitous marine bacterium.

## Introduction

Large-scale 16S rRNA gene sequence surveys of numerous oceans have identified the SAR11 lineage within the *Alphaproteobacteria* to be among the most ubiquitous bacterioplankton in the pelagic zone of marine ecosystems [1]. Members of this clade typically constitute between 25–50% of the total surface microbial community in coastal and open ocean environments [1]–[3]. With an estimated global population size of 2.4×10^28^ cells [1], these microbes are among the most successful organisms on the planet, and are actively involved in the cycling of nutrients in the ocean [4]–[7].

Based on genomic and physiological analyses of the few available pure cultures, *Candidatus* Pelagibacter ubique [4], [6], [8], [9], SAR11 cells have been shown to be photoheterotrophic, rely heavily on reduced sulphur compounds, and possess genes necessary for synthesizing essential vitamins and amino acids. However, the oxidation of simple sugars and the utilization of dissolved organic phosphates seem to be a special adaptation to the nutrient status of their respective marine environment [10], [11]. Numerous phylogenetic studies using marker genes such as the 16S rRNA gene [12], [13], the 16S-23S internal transcribed spacer (ITS) region [14], [15], proteorhodopsin gene [16]–[18], or a combination of multiple gene datasets [19]–[21] have also revealed a high diversity within members of this clade in marine environments. Sequences from these studies demonstrate stable phylogenetic clusters that are widespread across global scales [1], [22]–[24], exhibit distinct vertical distribution along the water column [2], [14], [25], and illustrate spatio-temporal peaks of abundance that correlate with various physicochemical conditions of their environment [3], [26]–[29].

The divergence into several phylogenetic clusters that are ubiquitous in similar environments implies the presence of “ecotypes” in populations of SAR11 [24], [30]. Ecotypes are groups of very closely related organisms with distinct physiological adaptations to the prevailing condition [31]. While an understanding of the environmental constraints (e.g., temperature, light availability, and nutrient gradients) that select for the success of one ecotype over the other is at an advanced stage for the cyanobacteria of the genus *Prochlorococcus* [32], knowledge of the respective micro-niches in SAR11 populations are still unclear. In spite of this, the combined analysis of the ITS and the corresponding 16S loci has provided a backbone to address questions on the macro- and micro-niches of potential ecotypes [14], [18], [23], [24], since they provide different levels of phylogenetic resolutions.

Our recent survey of bacterioplankton communities of the north-eastern sector of the Red Sea demonstrated that members of the SAR11 clade constituted two thirds of bacterial communities in the surface waters of this saline water body [33]. Phylogenetic analyses of this clade further indicated that the predominant SAR11 sequence-types, which constituted ∼50% of total 16S rRNA gene sequences, grouped within the Surface 1a and b clusters. Surprisingly, the Red Sea’s population of SAR11 closely resembled that in the Sargasso Sea in spite of the geographic distance and considerable differences in physicochemical conditions between these water bodies [34]–[36].

A remarkable feature of the Red Sea, which sets it apart from most other oligotrophic provinces of the world’s oceans, is the isothermal (∼22°C) and isohaline (40.6 psu) deep water mass (from 200 m to the bottom) that characterizes the entire basin of this ecosystem [35], [37]–[39]. Except for the Mediterranean Sea, which is connected to the Red Sea and has an average temperature of 14°C from roughly 300 m to the bottom [40], [41], temperature in most global oceans decreases with depth to 3–5°C below 500 m. A persistently high surface water temperature (i.e., ∼24°C in spring and up to 35°C in summer) coupled to prolonged thermal stratification and a slow turnover rate of the deep-sea water mass [42], [43] as is the case in the Red Sea, should have a significant impact on the composition and the flux of dissolved organic carbon (DOC). Consequently, we hypothesize that the assemblage of heterotrophic communities degrading DOC in the water column of the Red Sea, especially the deeper layers, should be different from other oligotrophic oceans with moderately lower temperatures.

Here, we exploited the heterogeneity of the ITS loci in conjunction with partial 16S rRNA genes to investigate the microdiversity of SAR11 [14], [15] in the water surface (10 m), the mesopelagic (200–700 m), and the bathypelagic (1500 m) zones of the Red Sea’s water column. This approach allowed for a robust linkage of 16S rRNA gene clusters (referred to here as subgroups) with ITS clusters (referred to here as phylotypes) of hitherto undescribed ecotypes. Publicly available metagenomic libraries provided an excellent means to compare the distribution of dominant “ecotypes” from four distinct marine ecosystems with a seasonally similar (summer) dataset from the Red Sea using blast-based searches.

## Results

### Diversity and Community Structure of 16S-23S ITS Sequences

We generated a total of 2,346 sequences that spanned the 16S-23S ITS region (∼1,200 bp long; 109–247 clones per library; Table 1) from water samples that had been collected at 10 m (*n* = 11) and four samples of a water-column (at 50, 200, 700, and 1,500 m depths; Figure S1A). The surface water samples spanned the coastal and open-ocean environments of the north-eastern sector of the Red Sea, whereas the water-column samples were obtained from a site in the central region. The diversity captured by the 16S rRNA gene-sequence fraction (∼700 bp) of the amplicons was three times higher in deep water samples than those from the surface; the same was true for the sampled species diversity as reflected by the Simpson’s Reciprocal Index (Table 1). At the same distance threshold (3%), the diversity covered by the ITS fragments (∼400 bp) was generally higher than that of the 16S rRNA gene. The overall diversity for all libraries was 10 times higher at the ITS level (975 OTUs) than at the 16S rRNA gene-level (98 OTUs). Also, in contrast to 16S rRNA gene sequences, surface water samples produced up to 23-folds the diversity observed in the deeper waters at the ITS level (Table 1), suggesting that the communities in the epipelagic zone have a much higher microdiversity.

**Table 1:** Number of sequences (no. seq) and the diversity estimates from partial 16S rRNA gene fractions (∼700 bp) and the related ITS fragments (∼400 bp) of clone libraries used in this study.

		16S rRNA gene fraction				ITS fraction				
Transect (T)^a^	No. seq	OTUs	Chao1^b^	(*1/D*)^c^	% GEC^d^	OTUs	Chao1^b^	(*1/D*)^c^	% GEC^d^	Div*_ITS_*/Div*_16S_* e
**Surface (10 m)**										
T1_C	159	6	7–43	1.93	98	110	225–522	124	47	18.3
T1_OP	154	7	7–30	2.12	97	119	276–658	203	37	17.0
T2_C	165	7	7–30	1.97	98	129	342–871	246	36	18.4
T2_OP	135	5	5–29	1.88	98	113	300–798	302	28	22.6
T3_C	153	5	5–13	2.11	99	107	176–341	159	50	21.4
T3_OP	141	10	17–115	1.86	94	118	411–1326	260	26	11.8
T4_C	168	6	7–43	1.84	98	139	488–1428	298	27	23.2
T4_OP	121	7	9–59	1.61	96	109	438–1628	484	17	15.6
T5_OP	136	10	17–115	1.90	94	90	186–488	94	49	9.0
T6_C	154	8	11–76	1.42	96	111	274–718	117	41	13.9
T6_OP	109	5	5–29	1.23	97	69	154–503	39	49	13.8
**Water column**										
50 m	247	8	8–14	2.20	99	128	200–368	106	68	16.0
200 m	218	36	42–93	5.71	91	134	231–436	121	57	3.7
700 m	144	26	43–242	5.82	89	62	80–173	45	78	2.4
1500 m	142	29	42–186	9.66	88	80	134–320	61	61	2.8

Operational taxonomic units (OTUs) defined at a 3% distance threshold.

a The letters “C” or “OP” after the transect number (i.e., T1_#) denote coast and open ocean environments, respectively (details in Table S1).

b Ranges of estimated richness indices are reported as the 95% confidence interval.

c Simpson’s Reciprocal Index; higher values of this index indicate a highly diverse community.

d Good’s estimated coverage provides information of how large a fraction of the OTUs have been sampled more than once.

e The ratio between the number of OTUs observed within ITS sequences (Div*_ITS_*) and that of the 16S rRNA gene fractions (Div*_16S_*).

The number of OTUs (at 3% sequence difference) that we retrieved from each individual sample at the 16S rRNA gene-level was accounted only by a few OTUs that ranged from 5–36 (Table 1). Although these also represented nearly the expected diversity as reflected by Good’s estimated coverage (88–99%), the species richness obtained from the related ITS sequences were at the lower end of the Chao1 estimates (maximum coverage of 78%). Therefore, the microdiversity within these samples would still be considered to be undersampled (Figure S2), which is consistent with the higher number of observed OTUs at the ITS level (69–134).

Using two independent phylogenetic measures – Phylogenetic (P) test and UniFrac metric – that are not biased by the redundancy of OTU-based analyses, we found that irrespective of the sample, ITS sequences gave a considerably higher phylogenetic diversity (i.e., number of unique branch lengths on a phylogenetic tree) than their 16S rRNA counterparts; 3–7 versus 1–2, as expected and shown previously [14], [23]. This again highlights differences in phylogenetic coverage between the two phylogenetic markers, and underscores the higher resolution of the ITS-based analyses.

UniFrac-based community clustering in conjunction with principle coordinate analysis (PCoA) revealed a strong clustering of SAR11 from deep waters (200, 700, and 1500 m) that was significantly different from that in 10- and 50-m samples for both the 16S rRNA gene and ITS fractions (P test <0.001; Figure S3). However, we found no significant differences in community composition between surface water samples from the north and central Red Sea, and between coastal and open-ocean environments (*P*>0.01, ANOSIM). This implies a homogeneous distribution of these SAR11 populations throughout the pelagic environment of the north-eastern part of this water body.

### 16S rRNA Gene-based Phylogeny

The terms “subgroup” (S) and “phylotype” (P) are used here respectively to describe branches within the SAR11 clade based on either the 16S rRNA gene tree or that constructed from the corresponding ITS sequences as defined by Brown et al. [24]. This nomenclature is based on the pioneer works of Garcia-Martinez and Rodriguez-Valera [14], Brown and Fuhrman [23], Morris et al. [26], and Carlson et al. [2].

**Figure 1:**
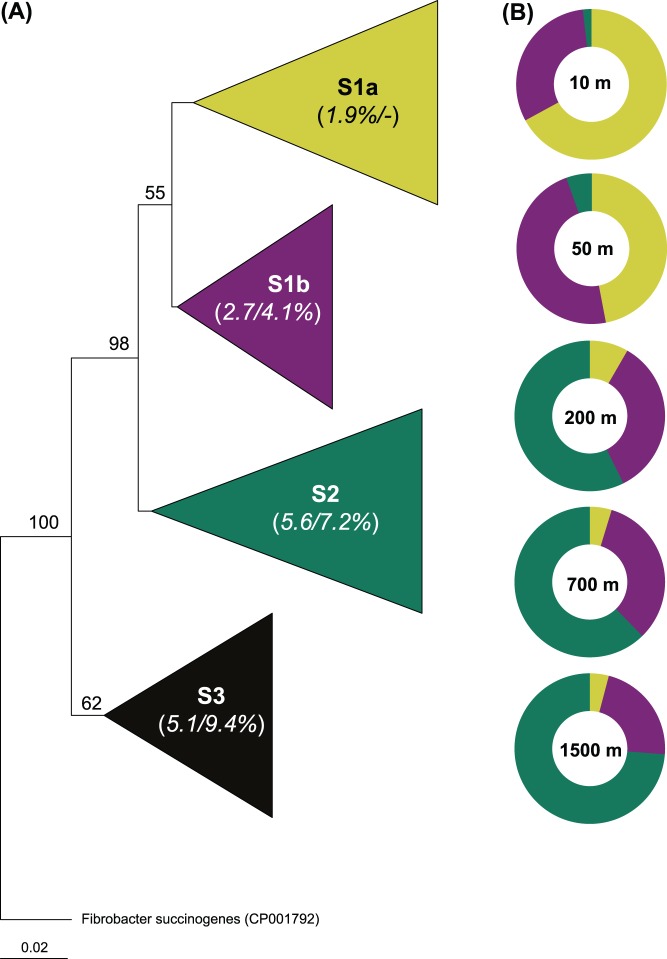
16S rRNA gene-based phylogenetic tree of subgroups and their relative abundances in the Red Sea’s water column. (A) Phylogram showing collapsed nodes (subgroups) that produced significant bootstraps with both neighbor-joining and maximum parsimony approaches in trees generated using PAUP. Numbers in bracket denote intra−/intergenetic divergence within each subgroup; the latter was calculated by comparing the rest with S1a. A detailed phylogenetic tree is provided as Figure S4. (B) Shows the shift in the relative abundances of each subgroup at different depths of the Red Sea’s water column.

Phylogenetic analysis of sequences that represented the 98 OTU-clusters of the 16S rRNA genes (97% sequence identity) generated in this study indicated that there were at least three SAR11 subgroups in the water column of the Red Sea (Figure 1 and S4). The average pairwise distance among sequences of a subgroup (i.e., intraspecific divergence) ranged from 2% to ∼6%, while the intergenetic divergence ranged from 4–10% (Figure 1), indicating that species within these groups are more diverse and distinct. In our entire library containing clonal sequences from all depths, the first and second most abundant OTUs clustered within S1a (52%) and S1b (31%), respectively, and shared a sequence identity of 94% (Table S2). Whereas OTU1 was closely related to three cultivated *Candidatus* Pelagibacter ubique strains of the same subgroup (HTCC1002, HTCC1062, and HTCC7211), OTU2 (RS_10M_S18_107) fell into subgroup S1b, which so far contains no cultured representatives, and shared sequence identities ranging from 92.7–93.3% to the above strains (Table S2). OTUs 3 to 5, which together accounted for 8.2% of all sequences, were largely found in deeper depths and belonged to the subgroup S2. These OTUs had a sequence identity of ∼96% to each other and <94% to the 16S rRNA genes of all presently available SAR11 strains (Table S2). Subgroup S3, which was not found in our Red Sea study, encompasses environmental sequences related to strains IMCC9063, isolated from seawater off the coast of Svalbard, Norway [9], and HIMB144, isolated from the coastal waters of Kanaohe Bay, Hawaii [44] along with environmental sequences from freshwater habitats (LD12; [28], [45], [46]).

The community profiles of surface water samples (10 m depth; *n* = 11) from the coastal and open-ocean environments of the north and central Red Sea were virtually identical (data not shown). As summarized in Figure 1B, they were all predominated by S1a (67%) and S1b (31%), whereas S2 accounted for the rest. However, at a depth of 50 m, while S1a still dominated the 16S rRNA gene library at this depth, S1b and S3 increased to 48% and 6% respectively. In the deeper waters (200–1500 m), the S2 subgroup dominated (52–74%; Figure 1B) over S1b (34–22%) and S1a (17–4%).

### Phylogeny of the Corresponding ITS Sequences

**Figure 2:**
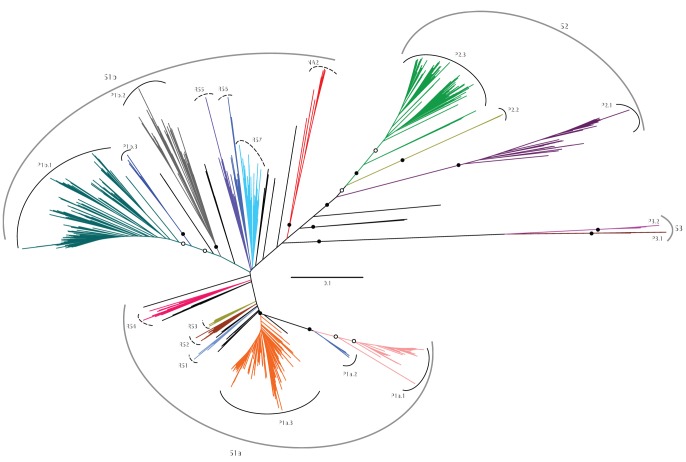
An unrooted ITS-based phylogenetic tree of phylotypes in the Red Sea’s water column. The tree was generated using 1,750 ITS sequences, which included representative from 975 OTU clusters derived from our clone sequences, that were derived from specific 16S subgroups (S). Branches with significant bootstrap support from trees inferred using both maximum parsimony and neighbor-joining algorithms, are shown with open (≥50%) or closed (≥70%) circles. Branches that constitute potentially novel phylotypes are shown with dashed lines. Sequence nametags were omitted for clarity; a detailed phylogenetic tree is provided as Figure S5.

ITS fragments extracted from our 16S rRNA clone libraries ranged in size from 368 to 450 bp and contained two tRNA genes encoding for alanine and isoleucine, which is characteristic of all described SAR11 ITS sequences so far [24]. Phylogenetic analysis of these sequences further revealed a much higher microdiversity than that already presented by their related 16S rRNA gene fractions (Figure 2 and S5). Here, unlike the 16S rRNA gene sequences, which were affiliated with three clusters (S1a, S1b, and S2), the corresponding ITS sequences were spread across 16 distinct phylotypes (Figure 2), including P1a.3, P1b.1, and P2.1, which were recently described as widespread in the pelagic waters of various tropical marine ecosystems [24]. As expected, four phylotypes that exclusively consist of sequences from cold marine environments (P1a.1 and P1a.2) or from brackish waters (P3.1 and P3.2) were completely absent in our clone libraries.

Interestingly, several potential deep-sea phylotypes that branch off at the bases of subgroups S1a (RS1–4) and S1b (R5–7 and NA2) and were absent in the global (surface water) survey of Brown et al. [24], were retrieved in our study. Their absence can be explained by the fact that the majority of sequences in these phylotypes are derived from deep-sea water environments like the Red Sea (200–1500 m; this study), the Mediterranean Sea (50–3000 m; [14], [40]), Sub-Arctic North Pacific (500 m; [47]), and North Atlantic (Greenland Sea, 2000 m; [40]). While these deeply branching phylotypes do not have strong bootstrap support, their levels of (intraspecific) divergence are comparable to those encountered among the subgroups, ranging from 9–22%. This not only supports the high level of diversity of sequences within these phylotypes, but also further suggests that even though relationship between these potential phylotypes is poorly resolved, the clustering within them is indeed strong. Moreover, as mentioned by Brown et al. [24], they include a few sequences from previous studies where the associated 16S rRNA gene fractions were lacking (in GenBank), and could therefore previously not be phylogenetically resolved at the subgroup level.

**Figure 3:**
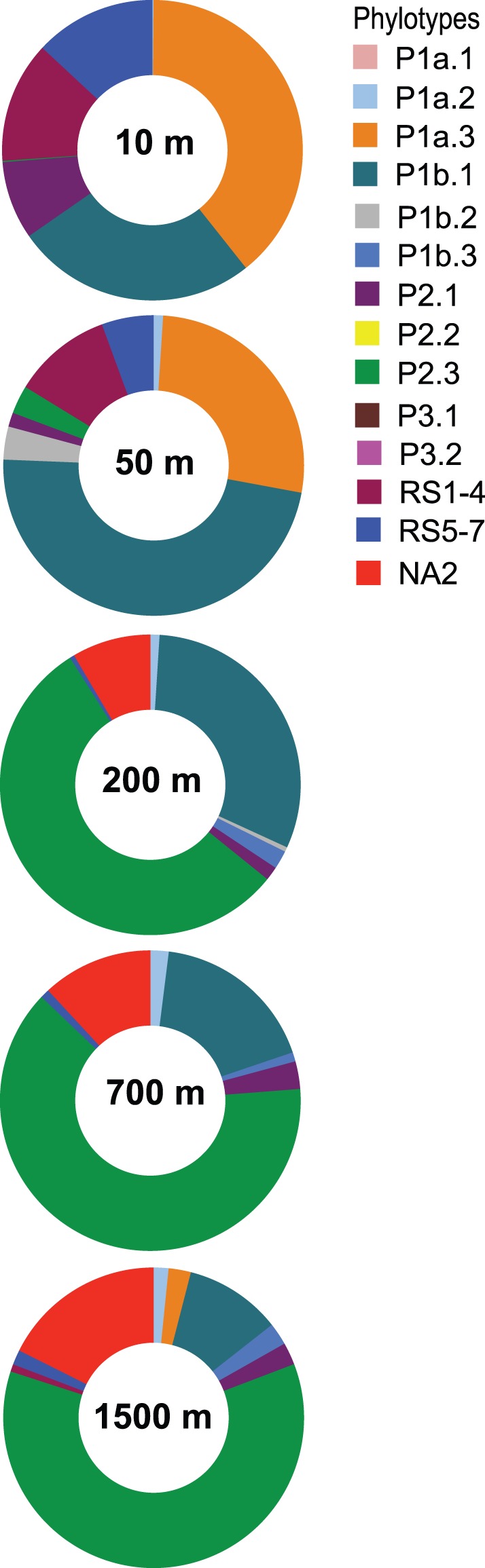
Depth-dependent shifts in phylotype abundances along the water column of the Red Sea. ITS sequences from each clone library were taxonomically assigned based on the annotated database presented in Figure 2. Results for the 10-m depth samples from coastal and open-ocean areas (*n* = 11) were summed together since no significant differences were observed.

In agreement with the depth-dependent shifts in 16S rRNA subgroups, we also found a clear vertical distribution of the major phylotypes in the water column of the Red Sea (Figure 3). While some phylotypes were predominating and occurred only in the upper euphotic zone (P1a.3, P1b.3, and RS1–4) or in the meso−/bathypelagic layer (P2.3 and NA2), sequences related with P1b.1 were distributed throughout the water column, albeit at different proportions. Their abundance was maximal at the 50-m deep chlorophyll maximum zone (Figure S1), constituting up to 48% of all sequences at this depth. In the mesopelagic (200 and 700 m) and bathypelagic (1500 m) zones, which are both characterized by a uniform *in situ* temperature of 22°C, we observed the predominance of phylotype P2.3 (55–63%), a decrease of P1b.1 (from 34 to 13%), and a moderate increase of NA2 with depth (Figure 3).

### Comparison to Other Water Masses

In order to determine the universality in the distribution and abundances of subgroups and phylotypes observed in our clone library-based results, we compared their distribution patterns in metagenomic libraries constructed from several samples that comprised seven distinct oceanic provinces (Table S3) including the Sargasso Sea (BATS), North Pacific Subtropicl Gyre (HOT), North-Western Atlantic Ocean (PRT), the Mediterranean Sea (three samples from different locations), and the Eastern Tropical South Pacific (ETSP, oxygen minimum zone) to a recent dataset from the Red Sea (summer, October 2008), using blast-based searches. The number of 16S rRNA gene sequences recruited from these metagenomes ranged from 27–519, whereas that of the ITS ranged from 20–133 (Table S3).

**Figure 4:**
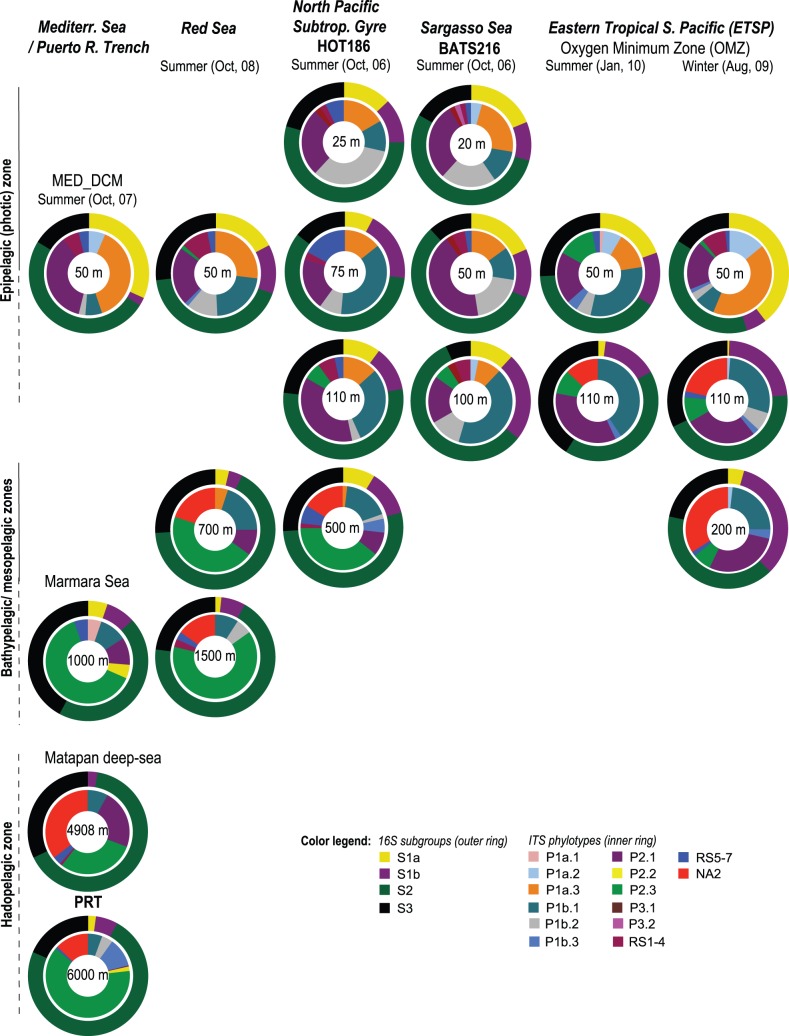
Relative shifts in the abundance of subgroups and phylotypes in the water column of five distinct oceans. The results are based on BLAST searches using metagenomic libraries against our annotated 16S and ITS datasets. Coloring details are as provided in Figures 2 and 3. Sample designations are provided in Table S3.

Consistent with our clone library-based results we observed that the recruited ITS sequences gave a better and refined picture of SAR11 distribution in the water columns of these geographically and environmentally distinct marine sites than the 16S rRNA genes (Figure 4). At the 16S level for example, sequences related to subgroup S2 dominated in nearly all samples. However, at the ITS level, the composition of phylotypes between the epipelagic zone and in the meso−/bathypelagic boundaries and between samples from BATS, HOT, MED, and the Red Sea and those from the ETSP were clearly different. Phylotypes P1b.1, P1b.3, and P2.1 were generally predominant in the epipelagic layer of nearly all the summer datasets (Figure 4), whereas P2.3 and NA2 were most conspicuous in the deeper layers.

**Figure 5:**
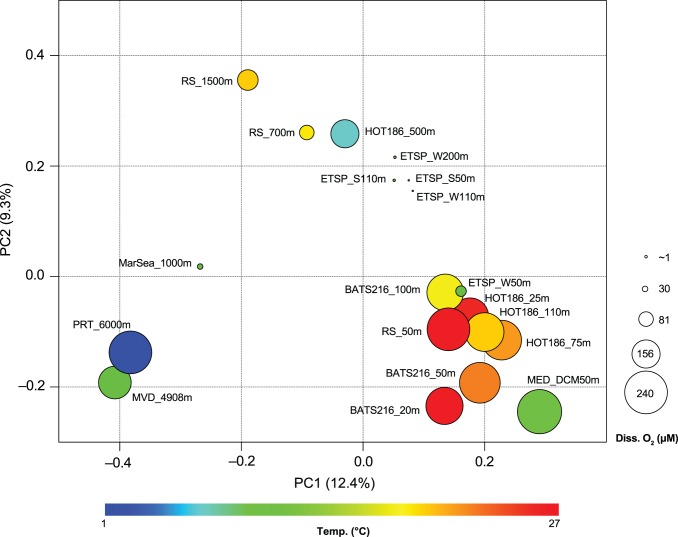
Principal coordinate analysis of the five distinct oceans based on the unweighted UniFrac distance matrix of phylotype sequences. Each circle is sized according to the concentration of dissolved O_2_ and color-coded according to the *in situ* seawater temperature. The percentage of variation explained by the plotted principal coordinate (PC) is indicated on the axes. Sample designations: RS, Red Sea; HOT, Hawaii Ocean Time Series; BATS, Bermuda Atlantic Time Series; ETPS, Eastern Tropical South Pacific; MED, Mediterranean Sea; MarSea, Marmara Sea; MVD, Matapan-Vavilov Deep; and PRT, Puerto-Rico Trench. More details are provided in Table S3.

Hierarchical clustering of UniFrac-based distances using principal coordinate analysis (PCoA) showed that irrespective of their season of collection (Figure 5), the SAR11 communities in samples from the ETSP significantly separated from those in BATS, HOT, MED, and the Red Sea (*P*<0.001, P Test; Table S4). This grouping was driven primarily by the depth of the water column (Spearman’s rank coefficient, *r* = ––0.77; *P*<0.001; PC1) and the concentration of dissolved O_2_, (*r* = –0.74; *P*<0.001; PC2), and moderately by Chlorophyll *a* (*r* = 0.63; *P*<0.01; PC1), and temperature (*r* = 0.54; *P*<0.05; PC1). These results show that while the specific physicochemical traits of the water column (e.g., less O_2_, low nutrients, and high temperature) play an important role in the vertical partitioning and distribution of SAR11, the populations in the Red Sea are not surprisingly different from those in other similar oceanic environments like BATS, HOT, and MED.

## Discussion

Phylogenetic comparisons of 16S rRNA genes have proven to be extremely useful for determining evolutionary relationships among prokaryotes from the domain level to that of closely related species and strains. Despite this, the evolutionary distances exhibited by 16S rRNA gene sequences of subgroups within the SAR11 clade (4–15% sequence identities; [14]) are in a range that is comparable to other well-characterized genera or even families [48]. However, even among cultivated strains of this bacterium that fall on the same branch of the 16S rRNA gene tree, there is sufficient physiological and genomic evidences that support clear phenotypic differences among these organisms [20], [21], [49]. The underlying populations of these genetically and physiologically distinct groups, also referred to as “ecotypes” [31], are usually differentially distributed along environmental gradients. While this phenomenon is well documented for the cyanobacteria of the genus *Prochlorococcus* [50], at a scale that has permitted its inclusion into large-scale oceanographic models [51], analogous studies for the ubiquitous marine SAR11 bacteria and a clear understanding of the ecological functions of the ecotypes are still lacking. Together with results from other environments, we present supporting evidence for combining 16S rRNA gene phylogenies with that of the related ITS loci, which has not only allowed us to correlate 16S rRNA subgroups to ITS phylotypes in the water column of the Red Sea, but also indicated that there might be different forces shaping the speciation of SAR11 lineages in different depths of the ocean.

### Vertical Partitioning of SAR11 in the Red Sea

The accumulating evidence from both our current and previous studies provide clear evidence that the SAR11 population in the Red Sea is highly diverse and consists of several clusters that are structured vertically along the water column. These results are consistent with reports from the Atlantic and Pacific Oceans [2], [3], [25], [26], suggesting that there is a shift in the ecological niches and functions of SAR11 populations between the highly mixed surface layer and the meso−/bathypelagic zones in many subtropical oceans. The distinct profiles of distribution among these subpopulations also emphasizes the assumption that the SAR11 clade consists of numerous ecotypes with potentially different metabolic and ecological properties that are ecosystem specific [17], [20], [30]. In the case of the Red Sea, the complete absence of sequences related with P1a.1 (includes, *Candidatus* Pelagibacter ubique HTCC1062) and P1a.2 in the water column of this body, implies the presence of “ecotypes” that have potentially adapted to the warm-water environment of the Red Sea; surface water temperature ranges from 24°C (winter) to 35°C (summer; [52]). Both of these phylotypes were shown to preferentially occur in cold and nutrient-replete coastal environments [4], [23]. Temperature and nutrient concentrations therefore seem to be important forces driving the speciation of these bacteria in the world’s oceans synonymous with the temperature-dependent adaptive radiation that has long been witnessed for cyanobacteria of the genus *Prochlorococcus* [32], [50], [53] and some marine archaea [54]–[56].

The microdiversity of SAR11 was twenty fold higher in the upper euphotic zone than in the deep-sea layers (Table 1). This phenomenon is most likely a consequence of the high recombination rates that have been observed among SAR11 cells [19], which would presumably be elevated by the higher cell densities in the upper layers of the ocean and the harsh and ever-changing mixed-layer environment, compared to the rather stable conditions in the deeper layers. This would give rise to periodic selections and subsequently to microbial speciation [57]. In the Red Sea, this mixed-layer environment is characterized by relatively high temperatures (up to 30°C in summer; Figure S1) and moderately high fluxes of desert dust (micronutrient) inputs [58]. Relatively harsh conditions such as these are expected to exert strong selection pressures by accelerating the turnover of DOC [59] and via the Aeolian introduction of minerals and toxic compounds into the water column [60], [61], subsequently impacting metabolic rates and promoting redox reactions (e.g., N_2_ fixation [58], [62] and carbon sequestration [63]), thereby affecting microbial population dynamics [64]. The adaptation of such populations may also be tied with predation pressure for example from phage-host interactions, which often lead to fluctuations of successful lineages, and contribute to high levels of host diversity [65]. Other factors like exposure to high levels of UV irradiations [37], [66], [67], may not only have deleterious effects on their activities [68]–[70], but potentially select for a range of genetic traits (e.g., enriched repertoire of DNA repair and light stress genes) and probably distinct phylotypes of SAR11. Whether the possession of a specific proteorhodopsin pigment [16], [18], [71] contributes to ecotype differentiation, as suggested by vertical profiles of PR families in other marine environments and the optical preferences in the case of cyanobacteria [27], [72], [73], remains to be investigated in the Red Sea.

One interesting outcome of our study is that the ratio between the microdiversity covered by ITS sequences and the “macrodiversity” represented by the 16S rRNA genes diminishes towards the meso- and bathypelagic zones (Table 1). Based on the isothermal and isohaline character of the deep-sea water mass of the Red Sea (Figure S1), which is also estimated to have a renewal exchange rate in the order of decades (∼72 years; [42], [43]), we interpret these results to indicate that the relatively stable conditions in the deeper layers allowed for the differentiation and development of specialized quintessential subpopulations of SAR11 divergent from those in the (mixed) surface-water layer. The large proportions of subgroup S2-related sequences (∼50%, at 200–1500 m) in both clone libraries (winter) and metagenomic (summer) datasets, and the moderately lower intragenetic distances of the corresponding ITS fragments (5–10%) support this argument. Moreover, the cell densities at such depths are typically one order of magnitude lower than at the surface layers, thus reducing the potential for recombination events and predation pressure. Whether our conclusions can be extended to other marine ecosystems with strong thermoclines like the Sargasso Sea, should be a subject of great ecological interest. Also, since our work only represents a snap shot of the microdiversity in the north-eastern sector of the Red Sea, it will be necessary to demonstrate the universality of our findings across the whole Red Sea basin. This is particularly important due to antagonistic temperature–salinity effects on the surface water and nutrient imports from the Indian Ocean into the southern Red Sea’s waters.

### Emerging Biogeographical Patterns

Despite numerous descriptions and evidence of the oceanic distribution, abundance, and activity of members of the SAR11 clade, the relationship between “ecotypes” and ecological niches occupied by these bacteria has remained unexplored owing to the paucity of representative strains or genomes covering the entire lineage of SAR11. However, cross-ecosystem comparisons of the Red Sea with three of the most comprehensively studied sites in the global oceans (HOT, BATS, and ETSP) provide insights on the potential effects of temperature, seasonality, depth, and ecosystem-specific traits on ecotype distribution along the water column. Phylotype P1a.2 was for example, only encountered in the Mediterranean Sea and the Eastern Tropical South Pacific (ETSP), where the respective water temperatures at depths of 50 and 110 m were lower (∼14.0°C) than the temperature range (20–27°C) across the same depths in BATS, HOT, and the Red Sea. Along with the recent findings of Brown et al. (2012), which were based on metagenomic datasets of various pelagic (marine) environments around the globe, our results underscore the exceptional differences in the population of SAR11 from tropical and temperate oceans, particularly the striking absence of phylotypes P1a.1 and P1a.2 in tropical marine environments. This is equivalent with a temperature-dependent pattern of distribution that has been described for bacterial assemblages in pelagic environments of marine ecosystems [33], [74], [75], or ecotypes of *Prochlorococcus* [32], [50], [51] and the SAR86 clade [76].

Vertical differentiation in phylotype composition is also evident in the water column of these distinct ecosystems (Red Sea, BATS, HOT, and ETSP). This is probably correlated with changes in seawater chemistry (e.g., nutrient and O_2_ concentration) that varies greatly along the water column and might be ecosystem specific. Nutrient concentrations (e.g., inorganic nitrogen and phosphate) at the HOT and BATS sites can be several orders of magnitude lower than those in the Red Sea [62], [77], whereas in the ETSP they can be considerably higher at the surface, but extremely diminished below the oxycline [78], [79]. Metagenomic-based comparative analyses also indicate that there is a large variation of phosphate uptake genes [29], [80] among SAR11 populations found in phosphate-depleted environments like the Sargasso Sea (0.2–1 nM DIP; [62]) compared to those with moderately high DIP concentrations (0.03–0.95 µM; [81]) like the Indian Ocean, which is closer to the Red Sea. Moreover, many phosphate acquisition genes are almost absent in coastal strains (HTCC1002 and HTCC1062; [29], [80]) belonging to phylotype P1a.1, which is absent in the Red Sea, but present in their open-ocean counterparts in P1a.3 (HTCC7211). Altogether, these results imply that there is a correlation between the physiological and genetic attributes of representative SAR11 strains within these phylotypes and the environmental conditions in their native marine environments [11], [82].

The total fluorescence in summer peaks at a much lower depth in the Red Sea (∼75 m; Figure S1), BATS (80–120; [30]), and HOT (∼110 m; [83]) compared to the ETSP (∼20 m; [84], [85]). Such strong differences in nutrient availability would also have implications on the population dynamics of heterotrophic communities like SAR11 [30], which thrive on a diverse array of dissolved organic compounds, including those derived from the metabolic activities of phytoplanktons [6], [7], [26]. While some of the phylotypes do seem to co-occur in the water column of these marine ecosystems, both the carbohydrate utilization patterns of cultivated *Candidatus* Pelagibacter ubique strains [11] and genomic evidences [21], [44] suggest that the distribution of phylotypes P1a.1 (HTCC1062) and P1a.3 (HTCC7211) might be linked with the productivity of the ocean.

A critical parameter that has often been overlooked by all previous studies is the effect of oxygen on the metabolism and populations dynamics of these bacteria. The pelagic water masses at ETSP are extremely O_2_-depleted (<1 µM below the oxycline, 50–100 m; [86], [87]), unlike those at BATS, HOT, MED, and the Red Sea sites, where the concentrations of dissolved O_2_ are relatively high (∼100–250 µM) from the surface to a depth of approximately 200 m. In line with this important difference we observed that SAR11 communities at ETSP were significantly distinct from the rest of the subtropical sites (Figure 5). In the waters of Red Sea and ETPS, phylotypes P2.3 and NA2 seem to preferentially occur in O_2_-deficient deep-sea water masses; ranging from <90 µM O_2_ in the Red Sea (700–1500 m; Figure S1) to <5 µM O_2_ in ETSP (from ∼110 m downwards; [85]). Although the specific metabolic capabilities that enable some members of this clade to thrive in O_2_-deficient waters remain unknown, metagenomic studies of ETSP reveal that transcripts encoding for proteins that catalyse the conversion of sulphur to sulphate (*aprA* gene), were not only abundant but highly expressed and mostly affiliated with SAR11 throughout the oxycline (32% of top hits; [87]). While all members of this clade are unable to perform dissimilatory sulphate reduction, making them heavily dependent on reduced sulphur compounds for growth [6], recent evidences from comparative genomics suggest the potential to assimilate sulphate in some strains [44]. Together with their ability to demethylate dimethysulfoniopropionate (DMSP; [88]), these findings suggests a role for some members of this clade in sulphur cycling of OMZs.

Collectively, our comparisons highlight striking differences in the population structure of SAR11 along different depths of the water column and also between subtropical provinces of the global ocean. Together with reports from previous studies, our results therefore reinforce the general concept that the physico-chemical constraints imposed by different marine ecosystems greatly impact on the population dynamics of this ubiquitous marine bacterium ([30]; and references therein), thereby giving rise to ecotypes in the todays oceans [24].

### Conclusions

This study evaluated the combined use of 16S rRNA gene and ITS loci to assess the microdiversity within lineages of the SAR11 clade in the Red Sea and several oceanic sites around the globe. In all cases, analyses based on the ITS region provided a significantly higher phylogenetic resolution and a refined community structure of SAR11 populations in the water column compared to the related 16S rRNA genes alone. As such, our study supports the growing use of ITS-based phylogenies to provide a scheme for classifying members of the SAR11 clade and to identify ecologically relevant trends of distribution in various disparate marine ecosystems. The high incidences of recombination events previously observed among native populations of this lineage and the high intraspecific diversity revealed by the ITS phylotypes implies a high degree of ecological differentiation within members of this clade, which may have contributed to their ecological success across different oceanic provinces. The fact that some phylotypes coexist only in certain marine environments reinforces the concept of niche partitioning that has been described for other abundant marine prokaryotes.

While the microbiology of the Red Sea is still poorly understood, it is becoming increasingly clear that the diversity of SAR11 in this saline water body is as high and phylogenetically diverse as that in other tropical oceans despite of its peculiar environmental traits. Temperature is also emerging as an agent driving the separation of tropical and temperate lineages of SAR11 across the global oceans. However, the influence of other variables such as nutrient availability and the concentration of dissolved O_2_, cannot be underestimated, as they may also be connected with seasonality and deep-water mixing events in some oceans. Because most of the currently known metabolic information of *Candidatus* Pelagibacter are based on strains that were isolated from cold marine environments, additional genomic information from subtropical lineages will be necessary to help address previously unresolved questions about the metabolic capacities and evolutionary histories of tropical lineages of this ubiquitous marine bacterium. Furthermore, the comprehensive ITS database from our study should facilitate the design of ecotype-specific qPCR primers targeting the ITS region, thus allowing extended and meaningful seasonal surveys of SAR11 across the oceans.

## Materials and Methods

### Sample Collection and DNA Extraction

Sea-surface water samples (10 m) from seven transects that covered the coast-to-open ocean environment of the Red Sea were collected during the 2^nd^ *R/V Aegaeo* WHOI-KAUST Red Sea Expedition in March 2010 (Table S1). The DNA from these samples was readily available from our recent study [33]. In addition, DNA extracts from four samples (50, 200, 700, and 1,500 m depths) collected from a single location in central Red Sea (21°20.76′N, 38°04.68′ E; same cruise as above) were kindly provided by Prof. Hamza El Dorry (American University of Cairo, Egypt), and collectively used to complete the microdiversity picture of SAR11 in the water column of the Red Sea.

### PCR, Cloning, and Sequencing

PCR amplification of the 16S-23S internal transcribed spacer (ITS) region of SAR11 was done using a forward primer specific for SAR11 16S rRNA genes, and a reverse primer specific for 23S rRNA genes of *Alphaproteobacteria* [14]. Each PCR reaction mixture contained: (final concentrations) 1× PCR buffer (New England Biolabs), dNTPs (100 µM), primers (1 µM), *Taq* DNA polymerase (2.5 U; Invitrogen), and 10 ng of template DNA. The PCRs were run with the following conditions: initial denaturation at 94°C for 2 min; followed by 30 cycles at 94°C for 20 s, 50°C for 20 s, 72°C for 2 min; and a final extension at 72°C for 10 min. Amplicons were quality-assessed on agarose gels prior to purification with the QIAquick purification kit (Qiagen, Hilden, Germany). Purified PCR products were then cloned into the pCR® 2.1 vector (Invitrogen) as per the manufacturers protocol. Colonies with the correct insert size were then bi-directionally sequenced with M13 primers on an ABI 3730×l DNA Analyzer in the Genomics Core Lab facility at KAUST (Thuwal, Saudi Arabia), and assembled using Geneious Pro software (v5.5; http://www.geneious.com/).

Pairwise alignments of all sequences were performed using Geneious aligner that implements a progressive pairwise algorithm for multiple alignments. Aligned sequences were then manually edited followed by the extraction of partial 16S rRNA gene fractions (∼700 bp) and their corresponding ITS regions (∼400 bp) from the global alignments based on primer sequences and an annotated SAR11-specific ITS database containing 862 sequences [24]. Chimeric sequences were checked using both Bellerophon in Greengenes (http://greengenes.lbl.gov/cgi-bin/nph-index.cgi) and ChimeraSlayer (http://microbiomeutil.sourceforge.net/), and removed prior to downstream analyses (25 out of 2371). The presence of tRNA genes within the ITS fragments were predicted using tRNASCAN-SE, version 1.21 (http://lowelab.ucsc.edu/tRNAscan-SE/).

### Diversity Estimates and Phylogenetic Analyses

Following the above procedures, we obtained a total of 2,346 high-quality sequences (∼1200 bp) of the 16S-23S ITS region. The 16S rRNA gene and ITS portions of these sequences were then aligned with ClustalW (as implemented in Geneious), and subsequently clustered into operational taxonomic units (OTUs) with an identity match of 97% using software packages in MOTHUR [89]. These 97%-identity clusters of OTUs were then used to compute diversity indices (Chao, Simpson’s Reciprocal Index, and Coverage). Alternatively, phylotype-based measures of community similarity (Phylodiversity; [90]) as well as phylogenetic-based measures (UniFrac metric; [91]) were applied to capture the genetic differences (alpha and beta diversities) between the 16S rRNA gene fragments and their corresponding ITS regions. Both of these estimates are based on a phylogenetic tree of all sampled sequences; the former calculates the total number of unique branch lengths in the tree while the later computes the branch lengths shared between sampled communities within the tree. Distance matrices generated using weighted UniFrac were also clustered into two-dimensional space by applying principal coordinate analysis (PCoA).

Sequences representing each OTU (98 and 975 respectively for 16S rRNA gene and ITS loci) were further employed to infer phylogenetic relationships with their closest best BLAST hits from NCBI (http://www.ncbi.nlm.nih.gov/). The 16S rRNA gene sequences (∼700 bp) were together aligned using the SINA web alignment tool (http://www.arb-silva.de/aligner/), imported into the ARB software package [92] containing a SILVA ver.111 non-redundant 16S rRNA reference database (http://www.arb-silva.de/download/arb-files/), manually corrected, and used to construct a phylogenetic tree using a distance-based neighbor-joining algorithm (1000 replications) implemented in PAUP (version 4.0). Maximum parsimony was also used to test the stability of tree topology.

For ITS sequences, pairwise alignments were performed in Geneious Pro based on the 657 base-pair alignment of Brown et al. [24]. After manual correction, all bases of these alignments were then used to infer relationships between sequences from the Red Sea and their closest neighbors using PAUP as explained above. Phylogenetic trees were visualized in FigTree (v1.3.1; http://tree.bio.ed.ac.uk/software/figtree/). All clonal 16S-23S rRNA gene sequences used for phylogenetic inferences have been deposited in GenBank under the accession numbers JQ991938–JQ992912.

### SAR11-specific ITS Surveys of Select Metagenomic Libraries

The abundance and distribution of SAR11 subgroups and phylotypes in different water column depths of other oceans was assessed by mining seven publicly available metagenomic data sets from the NCBI Short Reads Archive (Table S3), and three unpublished metagenomes from the Red Sea (Oct, 2008; Thompson et al., submitted; El Dory et al., unpublished). All 454 datasets were trimmed and quality checked using CLC Genomics Workbench with default settings. These metagenomes were then interrogated using Blastn for homologous sequences against our annotated 16S rRNA gene and ITS databases. Best matches to our query sequences were counted as those that had a sequence identity of >97% over a minimum length of 200 bp to the query sequence, bit score value of >40, and an expectation value of <10^–5^ (Table S3). The relative abundance of each subgroup or phylotype in a sample was then expressed as a percentage of all sequence counts recruited per sample. The statistical significance in the composition of pairs of libraries was calculated using the phylogenetic (P) test [93] based on a neighbour-joining tree of all samples as implemented in MOTHUR [89]. Unweighted UniFrac metric [91], which considers the absence or presence of lineages, was calculated from this tree and used to determine the community similarity among sites with PCoA as described above.

## Supporting Information

Figure S1 **Physicochemical parameters in the water column of central Red Sea.** Vertical profiles illustrate conditions that are typically present in spring (A), when the clone libraries were made. The summer profiles (B) from the metagenomic data that was used for comparison with similar data sets from other ecosystems was included to highlight seasonal differences between this two periods namely: (1) the deeper depth of the oxygen minimum in spring, (2) the surface water temperature increase in summer (by 4°C), and (3) stability and similarity of temperature and salinity conditions below 200 m.
(PDF)

Figure S2 **Rarefaction curves for (A) 16S rRNA gene sequences and (B) the related ITS fragments in our clone libraries.** For clarity, curves for the 10-m depth samples, represent the overall number of OTUs obtained for all surface water samples (*n* = 11; Table 1). Note the high number of observed OTU counts for the ITS loci in comparison to the related 16S rRNA genes among all samples.
(PDF)

Figure S3 **Principal coordinate analysis (PCoA) showing the clustering of SAR11 populations in the different samples based on the weighted UniFrac distance metric for both 16S rRNA genes and ITS sequences.** The percentage of variation explained by the plotted principal coordinates (PC) is indicated on the axes. Additional details for each sample are provided in Table 1. Basically the same clusters are found for both markers with deep-sea samples (200, 700 and 1500 m) always grouping together.
(PDF)

Figure S4 **Phylogeny of 16S rRNA genes generated in our study.** This tree provides additional phylogenetic details of sequences within each subgroup, which are depicted as collapsed nodes in Figure 1 including, S1a, S1b, and S2. Sequences from the Red Sea are highlighted in green; none of which were affiliated with S3. The tree was constructed from 98 representative OTUs (clustered at 3% sequence dissimilarity) and other related 16S rRNA gene sequences (∼700 bp) using the neighbor-joining approach with Jukes and Cantor correction in PAUP (Version 4.0b). Trees inferred using maximum parsimony gave virtually the same branching topology.
(PDF)

Figure S5 **Phylogenetic tree showing the position of the corresponding ITS fragments.** This tree provides additional phylogenetic details of the various phylotypes, which are depicted as in Figure 2. Sequences from our study are highlighted in green and include representative sequences (975 OTUs at 3% sequence dissimilarity; ∼400 bp positions) from this work that were used for phylogenetic inference based on neighbor-joining approach with Jukes and Cantor correction in PAUP (version 4.0b). None of our clone sequences were affiliated P1a.1 and P1a.b. *Prochlorococcus* sp. NC_005072 was used as an outgroup.
(PDF)

Table S1 **Environmental and physicochemical traits of seawater samples used for clone libraries in our study.**
(PDF)

Table S2 **Identity of the major OTUs in our clone libraries (with an abundance of >1%) based on the 16S rRNA gene, relative to each other and to cultivated strains within the SAR11 clade.**
(PDF)

Table S3 **Metagenomic libraries and their associated metadata that were used to recruit various SAR11-specific ITS reads and 16S rRNA gene sequences homologous to those in our annotated reference databases.**
(XLSX)

Table S4 **Sample pairs with significant differences in phylotype composition based on Phylogenetic/Parsimony (P) test.**
(PDF)
