# Correction: Combined Analyses of the ITS Loci and the Corresponding 16S rRNA Genes Reveal High Micro- and Macrodiversity of SAR11 Populations in the Red Sea

**DOI:** 10.1371/annotation/99cbcee6-fcc9-441b-a350-7073b3e0361e

**Published:** 2013-06-04

**Authors:** David Kamanda Ngugi, Ulrich Stingl

There was typographical error in the labeling of Figure 2. Please use the following link to access the correct version of Figure 2: 

**Figure:**
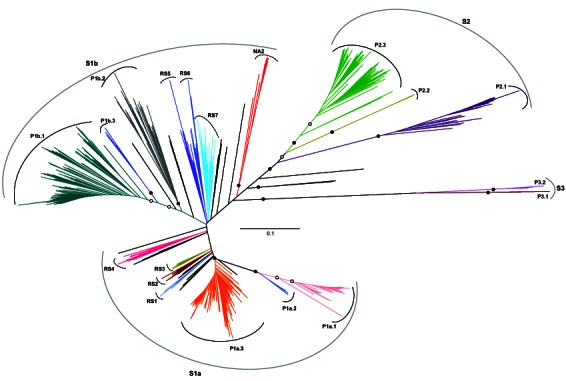


.

