# Supplementary figures and images for: Combined Analyses of the ITS Loci and the Corresponding 16S rRNA Genes Reveal High Micro- and Macrodiversity of SAR11 Populations in the Red Sea
Source: PLoS One. 2012 Nov 20;7(11):e50274. doi: 10.1371/journal.pone.0050274 (PMC3502338; doi:10.1371/journal.pone.0050274)

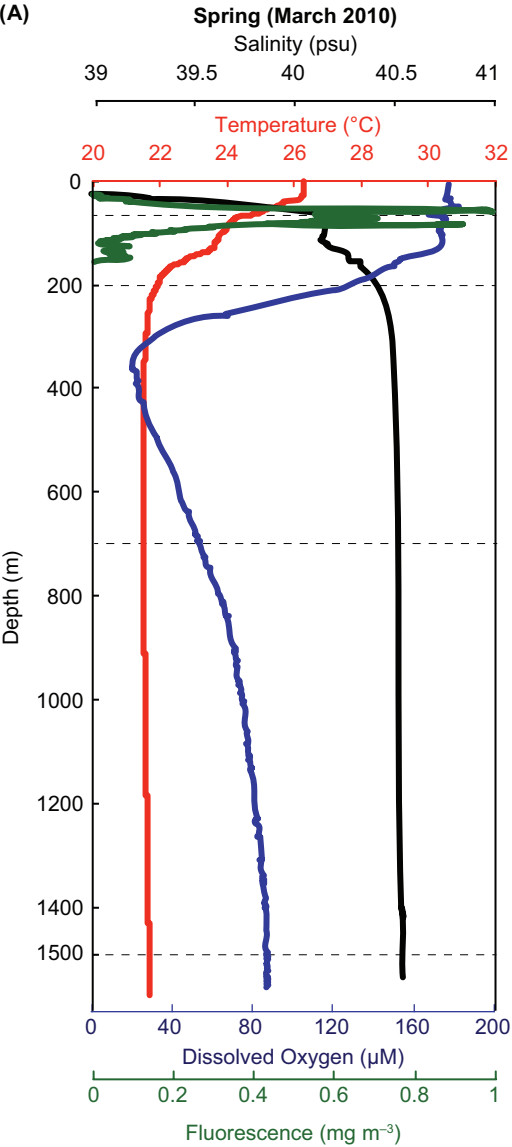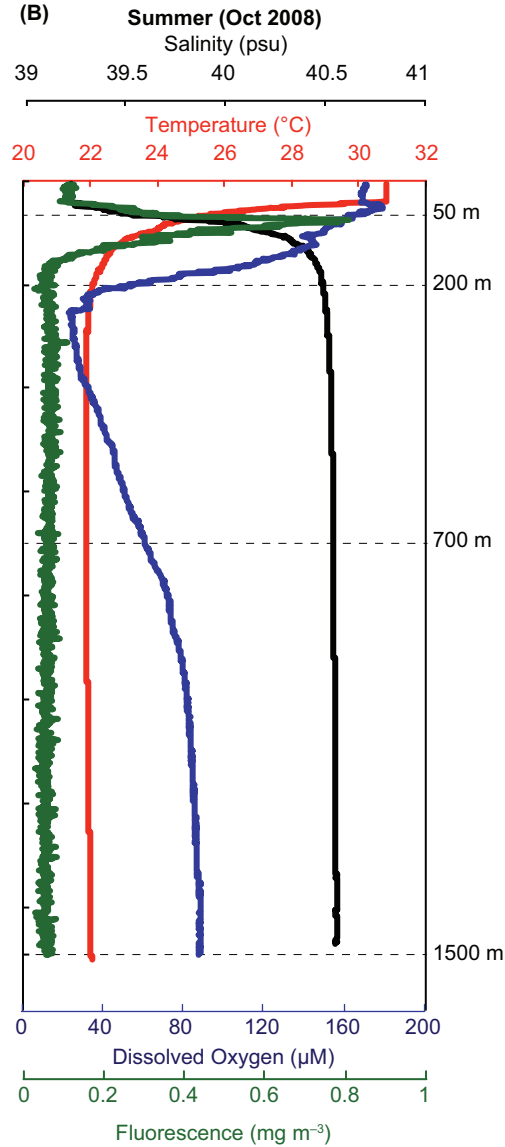

Supplement: Figure S1 — Physicochemical parameters in the water column of central Red Sea. Vertical profiles illustrate conditions that are typically present in spring (A), when the clone libraries were made. The summer profiles (B) from the metagenomic data that was used for comparison with similar data sets from other ecosystems was included to highlight seasonal differences between this two periods namely: (1) the deeper depth of the oxygen minimum in spring, (2) the surface water temperature increase in summer (by 4°C), and (3) stability and similarity of temperature and salinity conditions below 200 m. (PDF) [file pone.0050274.s001.pdf]

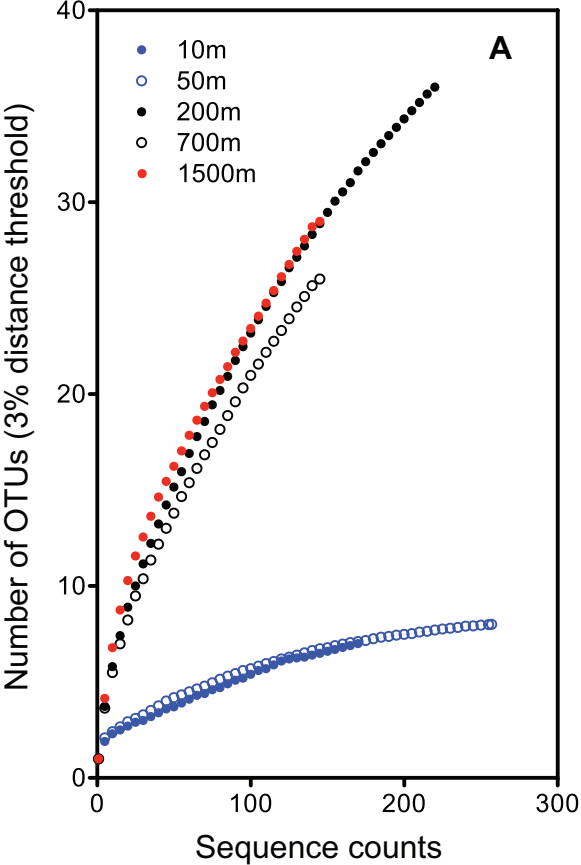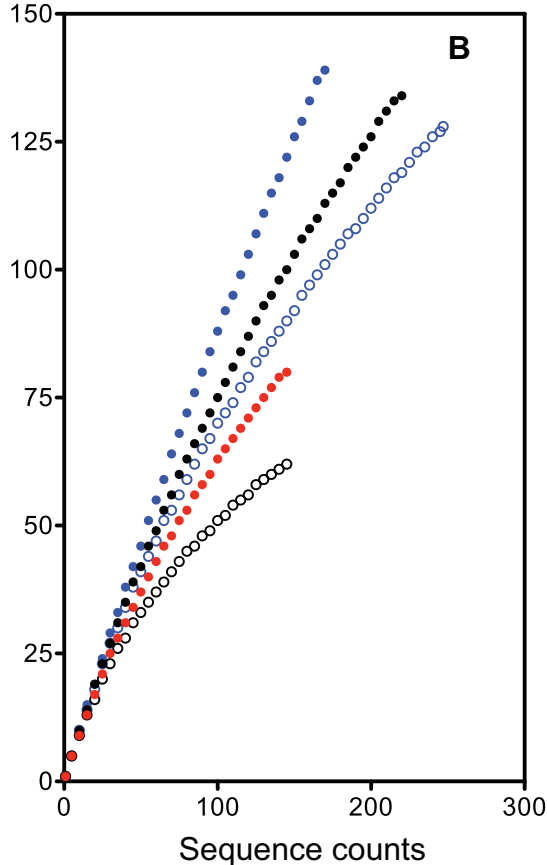

Supplement: Figure S2 — Rarefaction curves for (A) 16S rRNA gene sequences and (B) the related ITS fragments in our clone libraries. For clarity, curves for the 10-m depth samples, represent the overall number of OTUs obtained for all surface water samples (n = 11; Table 1). Note the high number of observed OTU counts for the ITS loci in comparison to the related 16S rRNA genes among all samples. (PDF) [file pone.0050274.s002.pdf]

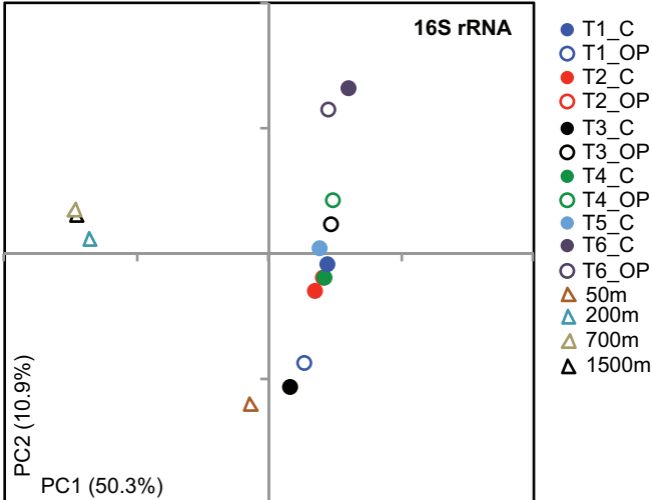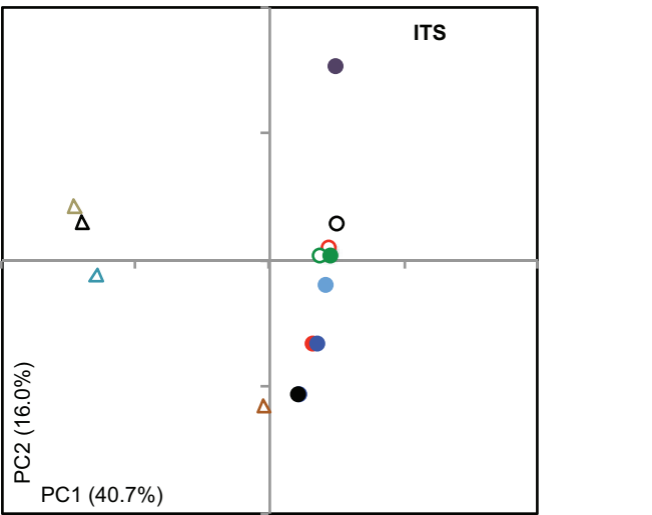

Supplement: Figure S3 — Principal coordinate analysis (PCoA) showing the clustering of SAR11 populations in the different samples based on the weighted UniFrac distance metric for both 16S rRNA genes and ITS sequences. The percentage of variation explained by the plotted principal coordinates (PC) is indicated on the axes. Additional details for each sample are provided in Table 1. Basically the same clusters are found for both markers with deep-sea samples (200, 700 and 1500 m) always grouping together. (PDF) [file pone.0050274.s003.pdf]

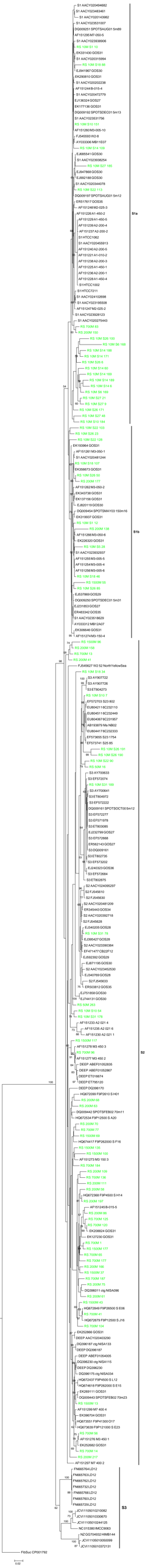

Supplement: Figure S4 — Phylogeny of 16S rRNA genes generated in our study. This tree provides additional phylogenetic details of sequences within each subgroup, which are depicted as collapsed nodes in Figure 1 including, S1a, S1b, and S2. Sequences from the Red Sea are highlighted in green; none of which were affiliated with S3. The tree was constructed from 98 representative OTUs (clustered at 3% sequence dissimilarity) and other related 16S rRNA gene sequences (∼700 bp) using the neighbor-joining approach with Jukes and Cantor correction in PAUP (Version 4.0b). Trees inferred using maximum parsimony gave virtually the same branching topology. (PDF) [file pone.0050274.s004.pdf]
