# Supplementary material for: Combined Analyses of the ITS Loci and the Corresponding 16S rRNA Genes Reveal High Micro- and Macrodiversity of SAR11 Populations in the Red Sea
Source: PLoS One. 2012 Nov 20;7(11):e50274. doi: 10.1371/journal.pone.0050274 (PMC3502338; doi:10.1371/journal.pone.0050274)
Supplement: Table S1 — Environmental and physicochemical traits of seawater samples used for clone libraries in our study. (PDF) [file pone.0050274.s006.pdf]

Table S1. Environmental and physico-chemical traits of seawater samples used for clone libraries in our study.

Transect [a]	Site / depth	Coordinates Lat. N / Long. E	Temp. (°C)	Salinity (psu)	Dissolved O_2 ($\mu m\ l^{-1}$)	Chl a [b] ($mg\ m^{-3}$)	PIC [b] ($mol\ m^{-3}$)	POC [c] ($mg\ m^{-3}$)
1	Coastal	27.82 / 35.08	24.23	40.00	178	0.150	0.000031	46.44
	Open ocean	27.44 / 34.82	23.80	40.04	178	0.133	0.000061	41.69
2	Coastal	27.25 / 35.62	24.40	39.89	176	0.125	0.000158	43.57
	Open ocean	26.91 / 35.14	23.92	39.99	177	0.131	0.000134	44.77
3	Coastal	25.89 / 36.49	25.05	39.72	174	0.158	0.000138	50.73
	Open ocean	25.66 / 35.78	25.35	39.41	173	0.145	0.000110	47.86
4	Coastal	25.17 / 36.89	25.32	39.48	174	0.153	0.000117	49.70
	Open ocean	24.96 / 36.35	25.55	39.42	173	0.148	0.000149	48.54
5	Open ocean	23.18 / 37.42	25.47	39.43	176	0.144	0.000128	47.75
6	Coastal	22.26 / 38.92	26.68	39.00	172	0.312	0.000171	90.54
	Open ocean	22.05 / 37.98	26.26	38.59	173	0.231	0.000166	66.63
	50 m	21.13 / 37.58	25.50	39.80	174.00	0.272		
	200 m	21.13 / 37.58	22.00	40.40	124.00	0.000		
	700 m	21.13 / 37.58	21.60	40.50	52.00	0.000		
	1500 m	21.13 / 37.58	21.70	40.60	85.00	0.000		

[a] For the transect samples, surface water was taken at 10-m depths.

[b] Satellite-based data averaged for the entire month of March 2010 (for details see, Ngugi et al., 2012).

PIC, paticulate inorganic carbon; POC, particulate organic carbon.
