# Supplementary material for: Combined Analyses of the ITS Loci and the Corresponding 16S rRNA Genes Reveal High Micro- and Macrodiversity of SAR11 Populations in the Red Sea
Source: PLoS One. 2012 Nov 20;7(11):e50274. doi: 10.1371/journal.pone.0050274 (PMC3502338; doi:10.1371/journal.pone.0050274)

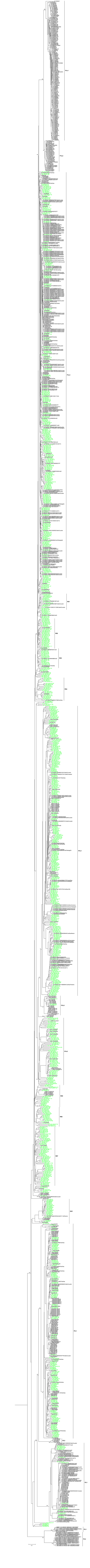

Supplement: Figure S5 — Phylogenetic tree showing the position of the corresponding ITS fragments. This tree provides additional phylogenetic details of the various phylotypes, which are depicted as in Figure 2. Sequences from our study are highlighted in green and include representative sequences (975 OTUs at 3% sequence dissimilarity; ∼400 bp positions) from this work that were used for phylogenetic inference based on neighbor-joining approach with Jukes and Cantor correction in PAUP (version 4.0b). None of our clone sequences were affiliated P1a.1 and P1a.b. Prochlorococcus sp. NC_005072 was used as an outgroup. (PDF) [file pone.0050274.s005.pdf]
