# Supplementary material for: Combined Analyses of the ITS Loci and the Corresponding 16S rRNA Genes Reveal High Micro- and Macrodiversity of SAR11 Populations in the Red Sea
Source: PLoS One. 2012 Nov 20;7(11):e50274. doi: 10.1371/journal.pone.0050274 (PMC3502338; doi:10.1371/journal.pone.0050274)
Supplement: Table S4 — Sample pairs with significant differences in phylotype composition based on Phylogenetic/Parsimony (P) test. (PDF) [file pone.0050274.s009.pdf]

Table S4. Sample pairs with significant differences in phylotype composition based on Phylogenetic/Parsimony (P) test.

Groups being compared (X-Y)	Parsimony Score	Significance (*P*)
BATS216_20m-ETSP_SUMMER_110m	25	<0.0010
BATS216_50m-ETSP_SUMMER_110m	22	<0.0010
BATS216_100m-ETSP_SUMMER_50m	18	<0.0010
BATS216_20m-ETSP_SUMMER_50m	21	<0.0010
BATS216_50m-ETSP_SUMMER_50m	19	<0.0010
BATS216_20m-ETSP_WINTER_110m	28	<0.0010
BATS216_50m-ETSP_WINTER_110m	25	<0.0010
BATS216_100m-ETSP_WINTER_200m	12	<0.0010
BATS216_20m-ETSP_WINTER_200m	14	<0.0010
ETSP_SUMMER_50m-ETSP_WINTER_200m	15	<0.0010
BATS216_100m-ETSP_WINTER_50m	16	<0.0010
BATS216_20m-ETSP_WINTER_50m	20	<0.0010
ETSP_SUMMER_110m-ETSP_WINTER_50m	19	<0.0010
ETSP_WINTER_110m-ETSP_WINTER_50m	21	<0.0010
ETSP_WINTER_200m-HOT186_110m	11	<0.0010
ETSP_WINTER_50m-HOT186_110m	14	<0.0010
ETSP_SUMMER_50m-HOT186_25m	15	<0.0010
BATS216_20m-HOT186_500m	24	<0.0010
BATS216_50m-HOT186_500m	21	<0.0010
ETSP_SUMMER_110m-HOT186_500m	22	<0.0010
ETSP_WINTER_200m-HOT186_500m	14	<0.0010
ETSP_WINTER_50m-HOT186_500m	18	<0.0010
ETSP_SUMMER_50m-HOT186_75m	18	<0.0010
BATS216_100m-MED_DCM50m	27	<0.0010
BATS216_20m-MED_DCM50m	38	<0.0010
BATS216_50m-MED_DCM50m	25	<0.0010
ETSP_SUMMER_110m-MED_DCM50m	35	<0.0010
ETSP_SUMMER_50m-MED_DCM50m	32	<0.0010
ETSP_WINTER_110m-MED_DCM50m	36	<0.0010
ETSP_WINTER_200m-MED_DCM50m	17	<0.0010
ETSP_WINTER_50m-MED_DCM50m	28	<0.0010
HOT186_110m-MED_DCM50m	20	<0.0010
HOT186_25m-MED_DCM50m	18	<0.0010
HOT186_500m-MED_DCM50m	25	<0.0010
HOT186_75m-MED_DCM50m	25	<0.0010
BATS216_100m-MVD_4908m	15	<0.0010
BATS216_20m-MVD_4908m	19	<0.0010
BATS216_50m-MVD_4908m	14	<0.0010
ETSP_SUMMER_110m-MVD_4908m	17	<0.0010
ETSP_SUMMER_50m-MVD_4908m	13	<0.0010
ETSP_WINTER_110m-MVD_4908m	22	<0.0010
ETSP_WINTER_200m-MVD_4908m	11	<0.0010
ETSP_WINTER_50m-MVD_4908m	12	<0.0010
HOT186_110m-MVD_4908m	13	<0.0010
HOT186_25m-MVD_4908m	12	<0.0010
HOT186_500m-MVD_4908m	15	<0.0010
HOT186_75m-MVD_4908m	11	<0.0010
MED_DCM50m-MVD_4908m	51	<0.0010
BATS216_100m-MarSea_1000m	10	<0.0010
BATS216_20m-MarSea_1000m	12	<0.0010
BATS216_50m-MarSea_1000m	9	<0.0010
ETSP_SUMMER_110m-MarSea_1000m	9	<0.0010
ETSP_SUMMER_50m-MarSea_1000m	11	<0.0010
ETSP_WINTER_110m-MarSea_1000m	11	<0.0010
ETSP_WINTER_200m-MarSea_1000m	8	<0.0010
ETSP_WINTER_50m-MarSea_1000m	9	<0.0010
HOT186_25m-MarSea_1000m	9	<0.0010
HOT186_75m-MarSea_1000m	8	<0.0010
MVD_4908m-MarSea_1000m	18	<0.0010
BATS216_100m-PRT_6000m	28	<0.0010
BATS216_20m-PRT_6000m	33	<0.0010
BATS216_50m-PRT_6000m	25	<0.0010
ETSP_SUMMER_110m-PRT_6000m	39	<0.0010
ETSP_SUMMER_50m-PRT_6000m	25	<0.0010
ETSP_WINTER_110m-PRT_6000m	45	<0.0010
ETSP_WINTER_200m-PRT_6000m	24	<0.0010
ETSP_WINTER_50m-PRT_6000m	21	<0.0010
HOT186_110m-PRT_6000m	19	<0.0010
HOT186_25m-PRT_6000m	17	<0.0010
HOT186_500m-PRT_6000m	34	<0.0010
HOT186_75m-PRT_6000m	25	<0.0010
MED_DCM50m-PRT_6000m	120	<0.0010
MVD_4908m-PRT_6000m	58	<0.0010
BATS216_100m-RS1500m	8	<0.0010
BATS216_20m-RS1500m	8	<0.0010
BATS216_50m-RS1500m	8	<0.0010
ETSP_SUMMER_50m-RS1500m	12	<0.0010
ETSP_WINTER_50m-RS1500m	10	<0.0010
HOT186_110m-RS1500m	8	<0.0010
HOT186_25m-RS1500m	8	<0.0010
HOT186_75m-RS1500m	8	<0.0010
MED_DCM50m-RS1500m	10	<0.0010
MVD_4908m-RS1500m	8	<0.0010
ETSP_SUMMER_110m-RS50m	32	<0.0010
MED_DCM50m-RS50m	41	<0.0010
MVD_4908m-RS50m	18	<0.0010
MarSea_1000m-RS50m	10	<0.0010
PRT_6000m-RS50m	39	<0.0010
RS1500m-RS50m	11	<0.0010

Only comparisons which were signficantly different are shown.
The phylotype composition for most samples differ from ETSP samples, which are characterized by moderately low temperature and suboxic conditions.
The second separation is between "surface" vs. deep-sea samples (e.g., RS1500m, PRT_6000m, MarSea_1000m, etc.).
