# Supplementary material for: Combined Analyses of the ITS Loci and the Corresponding 16S rRNA Genes Reveal High Micro- and Macrodiversity of SAR11 Populations in the Red Sea
Source: PLoS One. 2012 Nov 20;7(11):e50274. doi: 10.1371/journal.pone.0050274 (PMC3502338; doi:10.1371/journal.pone.0050274)
Supplement: Table S2 — Identity of the major OTUs in our clone libraries (with an abundance of >1%) based on the 16S rRNA gene, relative to each other and to cultivated strains within the SAR11 clade. (PDF) [file pone.0050274.s007.pdf]

Table S2. Identity of the major OTUs in our clone libraries (with an abundance of >1%) based on 16S rRNA gene, relative to each other and to cultivated strains within the SAR11 clade.

16S rRNA gene sequences / (abundance / *SAR11 subgroup* / occurrence) [a]	OTU1	OTU2	OTU3	OTU4	OTU5	*Candidatus* Pelagibacter ubique [b] HTCC1062	HTCC1002	HTCC7211	IMCC9063
OTU1 (52.2%; *S1a*; all depths)									
OTU2 (31.3%; *S1b*; all depths)	94.0								
OTU3 (3.7%; *S2*; all depths except 10 m)	92.6	91.7							
OTU4 (2.7%; *S2*; all depths except 10 m)	90.2	89.9	95.7						
OTU5 (1.8%; *S2*; all depths except 10 and 50 m)	93.1	91.9	96.9	94.4					
HTCC1062	98.5	93.3	92.6	90.8	93.1				
HTCC1002	98.4	93.2	92.5	90.6	92.9	99.8			
HTCC7211	98.7	92.7	92.9	90.2	92.8	98.9	98.7		
IMCC9063	85.7	82.7	86.6	86.0	86.2	85.9	85.7	86.3	
HIMB144	85.4	84.2	85.5	85.2	85.0	85.4	85.2	84.9	90.6

[a] The phylogenetic positions of sequence clusters represented by OTUs **1** (*RS_10M_S10_151*), **2** (*RS_10M_S18_107*), **3** (*RS_1500M_13*), **4** (*RS_700M_1*), and **5** (*RS_700M_96*), are provided in Figure S2.

[b] At 16S rRNA gene level, HTCC1062, HTCC1002, and HTCC7211 belong to subgroup S1a while IMCC9063 and HIMB144 belong to subgroup 3 (Figure S2).
